# Effects of Yishen Pinggan Recipe on Renal Protection and NF-*κ*B Signaling Pathway in Spontaneously Hypertensive Rats

**DOI:** 10.1155/2016/6435040

**Published:** 2016-03-16

**Authors:** Guodong Luo, Xiying Zhu, Zhongxiang Gao, Huaxun Ge, Yang Yu, Yuanyuan Guo, Jian-Pu Zheng, Longmin Liu

**Affiliations:** ^1^Department of Traditional Chinese Medicine, Putuo Hospital, Shanghai University of Traditional Chinese Medicine, Shanghai 200062, China; ^2^Emergency Department, Putuo Hospital, Shanghai University of Traditional Chinese Medicine, Shanghai 200062, China; ^3^Central Laboratory and Department of Cardiology, Putuo Hospital, Shanghai University of Traditional Chinese Medicine, Shanghai 200062, China

## Abstract

Inflammation is an important etiological factor of hypertensive renal damage. The effects of Yishen Pinggan Recipe (YPR) on urine microalbumin, histology, and NF-*κ*B/P65, I*κ*B-*α*, IL-1*β*, IL-6, and TNF-*α* in renal tissues were evaluated in SHR to explore the mechanism of its renal protection in hypertensive renal damage. The SBP of 12-week-old SHR was 192.41 ± 3.93 mmHg and DBP was 142.38 ± 5.79 mmHg. Without treatment, the 24-week-old SHRs' SBP was 196.96 ± 3.77 mmHg and DBP was 146.08 ± 4.82 mmHg. After the 12-week-old SHR were administered YPR for 12 weeks, the rats' SBP was 161.45 ± 7.57 mmHg and DBP was 117.21 ± 5.17 mmHg; YPR could lower blood pressure in SHR. And renal function damage was observed in 24-week-old SHR without treatment, manifested as urine protein and morphological changes which could be inhibited by YPR. In addition, YPR could reduce the expression of inflammatory cytokines (IL-1*β*, IL-6, and TNF-*α*) in kidneys. It could also inhibit the nuclear translocation of NF-*κ*B p65 and degradation of I*κ*B-*α* in renal cells, indicating that the NF-*κ*B signaling pathway was inhibited by YPR. Finally, the study suggests that YPR could significantly improve the renal function in SHR. The mechanism could be attributed to its inhibition of renal NF-*κ*B signaling pathway and inflammation.

## 1. Introduction

Kidney is one of the major target organs affected by hypertension. Forty-two percent of the primary hypertension patients not treated develop into kidney sclerosis, and about 10% die from renal failure [1, 2]. In addition, about 28% of end-stage renal diseases are related to hypertensive renal damage. Therefore, it is significant to prevent and treat the renal damage caused by hypertension.

In clinical practice, angiotensin-converting enzyme inhibitors (ACEI), angiotensin II receptor blockers (ARB), calcium-antagonists (CCB), and diuretic agents are employed to prevent and treat hypertension and its renal damage. But the results are unsatisfactory. The number of patients who developed into chronic renal dysfunction due to hypertensive renal damage is rising rapidly. Therefore, it is urgent to explore new prevention and treatment methods.

Traditional Chinese medicine (TCM) and traditional herbs are beneficial for hypertensive renal damage in both symptoms and causes. TCM can lower the blood pressure and protect the kidney as an efficient and practical means of prevention [3–5]. The Yishen Pinggan Recipe (YPR) is a compound prepared from tradition Chinese herbs used for treating hypertension. It is composed of prepared* Rehmannia root, Eucommia ulmoides, Mulberry parasitism*, Radix cyathulae,* Apocynum*, Puerarin, and* Uncaria*. It has been revealed in previous studies [6] that YPR could not only lower the blood pressure of the patients with early stage hypertensive renal damage but also reduce the urine microalbumin. It indicates that YPR has protective effects on renal functions, but the mechanism is still unclear.

In the present study, SHR was used to observe the influence of YPR on the inflammation factor (IL-6, IL-1 beta, and TNF alpha) and the activityof NF-*κ*B signaling pathway and explore the renal protective effects of YPR and the underlying mechanisms. It is to provide experimental basis for clinical application.

## 2. Materials and Methods

### 2.1. Experimental Agents

A YPR decoction consisting of seven herbs (*prepared Rehmannia root 30 g, Eucommia ulmoides 15 g, Mulberry parasitism 30 g, *Radix cyathulae* 15 g, Apocynum 30 g, *Puerarin* 15 g, and Uncaria 15 g*) was mixed in 300 mL of distilled water and extracted in a ceramic clay pot at 120°C. After boiling, it was extracted at 100°C for 30 min. Then 100 mL of the water extract was filtered through a sieve. Each of herbs was weighed according to the proportion and decocted until each milliliter contains 1.5 g drug. All herbs and decoctions were provided by the TCM pharmacy of the Putuo Hospital, Shanghai University of Traditional Chinese Medicine. Benazepril (Bena) was purchased from Novartis Pharmaceutical (Beijing, China).

Rats urine mAlb kits and urine *α*1-MG ELISA kits (XiTang Biotech Company Limited, Shanghai, China); rats TNF-*α*, IL-6, and IL-1 immunohistochemistry kits (Bohe Hengmai Biotech Company Limited, Shanghai, China); antibodies of NF-*κ*B p65, I*κ*B*α*, and GAPDH (Cell Signaling, Boston, USA); nuclear and cytoplasmic proteins extraction kits, BCA protein quantity kits, and RIPA lysate (Beyotime Institute of Biotechnology, Nanjing, China); rabbit anti-rat Histone H3.1 polyclonal antibodies (Signalway Antibody, Maryland, USA); and Luminescence agent (Millipore Corporation, Billerica, USA) were used.

### 2.2. Experimental Animals

Every 7 rats were housed in standard cages with controlled temperature (22 ± 2°C) and a 12 : 12 h light/dark cycle and received standard diet and water ad libitum. The care of rats was conducted in accordance with the national guidelines. We tried our best to minimize the rat's suffering. The rats were acclimatised to the metabolic cages. All the SHRs and WKY rats were purchased from Shanghai Slack Laboratory Animal Co., Ltd. (Shanghai, China). Twenty-one male SHR (SPF, 12-week-old, 250 g–280 g) were randomly divided into three groups: YPR group, Bena group, SHR group, with seven in each group. Another seven male WKY rats (12-week-old) were used as the normal control. The rats in YPR group were given YPR decoction 15 g/kg [7] with gavage beginning at 12 weeks of age. Rats in the Bena group were given benazepril water solution 1 mg/kg. Rats in the SHR group and WKY group were treated with equal amount of distilled water. All the administrations with gavage were given once a day and lasted for 12 weeks. The rats were used at 24 weeks of age.

### 2.3. Blood Pressure Measurement

Using tail-cuff method, the blood pressure of each rat was monitored three times continuously one day before administration and after 12-week administration by ALC noninvasive blood pressure meter (Alcott Biotech Co., Ltd., Shanghai, China). The rats were acclimatised to the tail-cuff blood pressure monitoring system before experiment. The average value was calculated.

### 2.4. Urine mAlb and *α*1-MG Measurement

Twenty-four-hour urine of rats was collected by metabolic cage after 12 weeks of administration. Rats urine mAlb and *α*1-MG were tested by ELISA.

### 2.5. Renal Histology Observation

After 12-week intervention, under intraperitoneal anesthesia with pentobarbital sodium (0.1 g/kg body weight) the abdominal cavity of rats was opened. The abdominal organs were identified, the left kidney was removed quickly, the renal capsule was stripped, and the kidney was cut transversely and submerged into 10% formalin for fixation. Afterwards the right kidney was harvested and placed on ice, the renal capsule was stripped, and three to four renal tissue cubes about 1 mm^3^ from the cortex were obtained and placed into glutaraldehyde for electron microscopic examination. The kidney was fixed in formalin for 12 h, dehydrated in gradient by alcohol, cleared by dimethyl benzene, and embedded with paraffin. They were cut with an microtome into 4 *μ*m slices and stained with HE automatically by pathological staining machine (ST 5010, Feica Company, Germany). After HE staining, the slides were observed by an electron microscope (OLYMPUS, Japan). The electron microscopic slides were observed and recorded by a transmission electron microscope (Philips Electron Microscopy, Netherlands).

### 2.6. Immunohistochemical Examinations of Renal Inflammatory Factors

The two-step immunohistochemical assay was employed. Sections (4 *μ*m) were deparaffinized and hydrated, blocked with 5% BSA after microwave heating, and incubated with 50 *μ*L primary antibodies solution (1 : 100) for 2 hours at 37°C. After the slides were washed with PBS, HRP labelling antibodies were added and cells were incubated for 1 hour at 37°C. After washing with PBS, the DAB solution was added for coloration under microscopy. Counterstained with hematoxylin after reaction termination, the slides were routinely dehydrated, cleared, and sealed. The primary antibody was replaced by PBS for negative control. The cells with buffy signaling particles and signal patches were positive, while the noncolored cells were negative. Each section was photographed 5 times using image module software under the magnification of microscope of 400 times. The IOD value of the images was calculated with the Image-Pro Plus 6.0 software.

### 2.7. Western Blotting

Nuclear proteins were extracted with the nuclear and cytoplasmic protein extraction kits (Beyotime, Jiangsu, China). Preparation of tissue extracts: we mix lysis buffer A (10 mmol/L Hepes with pH 7.9, 10 mmol/L KCl, 0.1 mmol/L EDTA, 1 mmol/L dithiothreitol, 0.4% Igepal CA-630, 5 *μ*mol/L leupeptin, 2 *μ*mol/L pepstatin A, 1 *μ*mol/L aprotinin, and 1 mmol/L phenylmethylsulfonyl fluoride) and nuclear extraction buffer B (20 mmol/L Hepes with pH 7.9, 0.4 mol/L NaCl, 1 mmol/L EDTA, 1 mmol/L dithiothreitol, and 1 mmol/L phenylmethylsulfonyl fluoride). And the proportion of them was 20 : 1. Then, we added PMSF to them. The kidney tissue was cut into very small pieces as far as possible. We mix tissue extracts and kidney tissue (the proportion: 100 uL tissue extracts and 30 mg kidney tissue) in the glass homogenizer and the ice bath for 15 min. Then, the homogenate was centrifuged at 1500 ×g for 5 min at 4°C and the precipitate was left. The precipitate was harvested and resuspended in 200 *μ*L lysis buffer A. Following a violent vortex, the obtained lysates were incubated for 10 min on ice and centrifuged at 12000 ×g for 5 min at 4°C. The supernatant, consisting of the cytoplasmic fraction, was aliquoted for analysis. The pellets were resuspended in 50 *μ*L nuclear extraction buffer B and then agitated for 30 min at 4°C. After centrifugation at 12000 ×g for 10 min, the supernatant containing nuclear extracts was collected. The total cellular proteins were extracted with RIPA lysis buffer (Beyotime, Jiangsu, China). The BCA protein quantity kits (Beyotime, Jiangsu, China) and the ultraviolet spectrophotometer were adopted to draw the standard curve of the protein concentration. All samples were stored at −80°C until further analysis.

After 40 *μ*g protein extraction was boiled for 5 minutes, the protein was transferred to the nitrocellulose membrane after electrophoresis and the membrane was blocked with 5% skim milk TBST solution at room temperature. After washing the membrane was incubated with primary antibody solution (1 : 1000) overnight at 4°C. After washing on the next day, the membrane was incubated with HRP labelling antibody solution (1 : 1000) for 2 hours at room temperature. After washing the membrane was visualized using ECL luminescence liquid. Using GAPDH and Histone H3.1 as protein sample volume for comparison, the odd ratio of NF-*κ*B p65 and I*κ*B*α* to GAPDH and Histone H3.1 was obtained for analysis.

### 2.8. Statistical Analysis

All the data were analyzed by SPSS 18.0 software. The experimental results were presented as $\overset{-}{x}\pm sd$. Results between groups were compared with single factor variance analysis (ANOVA tests). The LSD test was conducted with homogeneity of variance while with heterogeneity of variance Dunnett's *T*3 test was used. *P* < 0.05 was considered statistically significant.

## 3. Results

### 3.1. Influence of YPR on Blood Pressure

As shown in Table 1, the blood pressure of SHR rats was significantly higher than that of WKY rats. After the administration of YPR, both the systolic and diastolic blood pressure decreased significantly (*P* < 0.05). Although the effect of YPR was weaker compared with ACEI benazepril, it suggested that YPR could lower the blood pressure.

### 3.2. Influence of YPR on Urine Proteins

**Figure 1:**
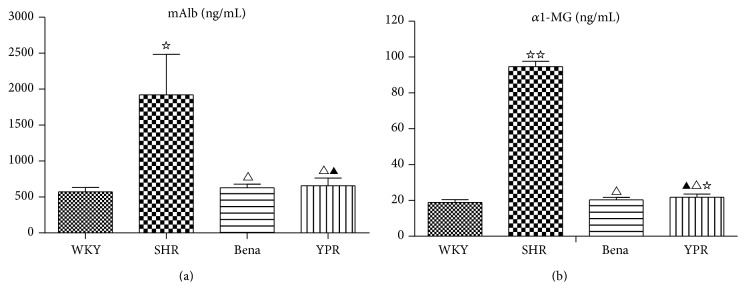
Influence of YPR on urine mAIb (a) and *α*1-MG (b) (*n* = 7). (a) Compared with the WKY group ^☆^ *P* < 0.01; compared with the SHR group ^△^ *P* < 0.01; compared with the Bena group ^▲^ *P* > 0.05. (b) Compared with the WKY group ^☆☆^ *P* < 0.01, ^☆^ *P* < 0.05; compared with the SHR group ^△^ *P* < 0.01; compared with the Bena group ^▲^ *P* > 0.05.

In comparison with WKY rats, mAlb and *α*_1_-MG in SHR urine were significantly increased. YPR could significantly reduce the levels of mAlb and *α*1-MG in SHR urine. The effect was similar to ACEI benazepril (Figures 1(a) and 1(b)), indicating that YPR was renally protective.

### 3.3. Influence of YPR on Renal Histological Manifestations

**Figure 2:**
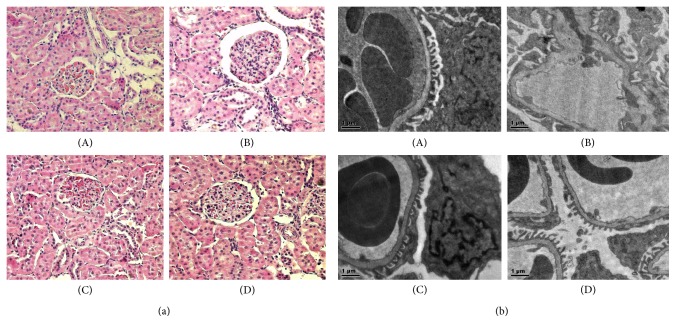
Influence of YPR on renal histologic manifestations. (a) HE staining under light microscopy (×200): (A) WKY group, (B) SHR group, (C) Bena group, and (D) YPR group. In the SHR group, glomeruli showed hyperemia, focal tubular epithelial cells were swollen with inflammatory cells infiltration, and partial arterioles showed wall thickening. No abnormality was found in the WKY group, Bena group, and YPR group. (b) HE staining under electron microscopy (×8200): (A) WKY group, (B) SHR group, (C) Bena group, and (D) YPR group. In the SHR group, there was basal membrane thickening, and focal foot processes were fused and disarranged. In the WHY, Bena, and YPR groups, no abnormalities were found.

Light microscopic examinations showed that, compared with WKY group, the mesangial cells underwent mild hyperplasia, focal renal tubular epithelial cells became swollen, inflammatory cell infiltrated, and arteriole and small arteries vessel walls presented hypertrophy. After the administration of YPR or benazepril, the mesangial region showed no hyperplasia and renal capsule did not expand. Obvious abnormalities were not found in renal tubules and significant inflammatory cell infiltration was not observed. No small artery thickening and hyalinization were recorded (Figure 2(a)).

The results of electron microscopic observations indicated that, compared with WKY group, the mesangial region in the SHR group underwent mild hyperplasia. The basement membrane became thickened, partial foot processes were fused and disarranged, and capillary loops reduced and collapsed. The mesangial region in the YPR and benazepril group was normal. No basement membrane thickening was observed and foot processes showed good shape and regular arrangement (Figure 2(b)).

### 3.4. Influence of YPR on the Levels of Inflammatory Cytokines in Renal Tissues

**Figure 3:**
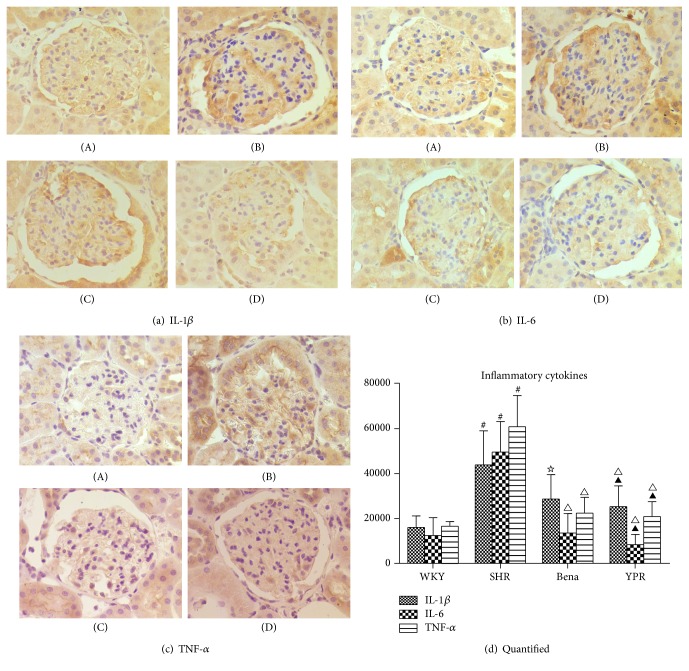
Influence of YPR on expressions of inflammatory cytokines including IL-1*β* (a), IL-6 (b), and TNF-*α* (c). (a), (b), and (c) are immunohistologic results of IL-1*β*, IL-6, and TNF-*α*: (A) WKY group, (B) SHR group, (C) Bena group, and (D) YPR group. (d) Compared with the WKY group ^#^ *P* < 0.01; compared with the SHR group ^△^ *P* < 0.01, ^☆^ *P* < 0.05; compared with the Bena group ^▲^ *P* > 0.05; compared with the WKY group, in the SHR group, expression levels of IL-1*β*, IL-6, and TNF-*α* in the renal tissues were significantly increased. In the YPR group, IL-1*β*, IL-6, and TNF-*α* expression levels in renal tissues were significantly decreased and no significant difference was found when compared with the WKY group.

Cytokines IL-1*β*, IL-6, and TNF-*α* play a significant role in the occurrence and development of hypertensive nephropathy [8, 9]. This study revealed that inflammatory cytokines IL-1*β*, IL-6, and TNF-*α* in SHR renal tissues were significantly higher than those in WKY rats. Traditional Chinese medicine YPR could significantly lower the level of inflammatory cytokines in renal tissue of SHR (Figure 3), indicating that it could ameliorate renal inflammation.

### 3.5. Influence of YPR on the Activity of NF-*κ*B in Renal Tissues

As an important inflammatory signal, the activation of NF-*κ*B could induce expressions of many cytokines, such as IL-1*β*, IL-6, and TNF-*α*. Moreover, the NF-*κ*B signaling activation in renal cells was positively correlated with inflammatory cytokines upregulation and mononuclear cell infiltration [10].

**Figure 4:**
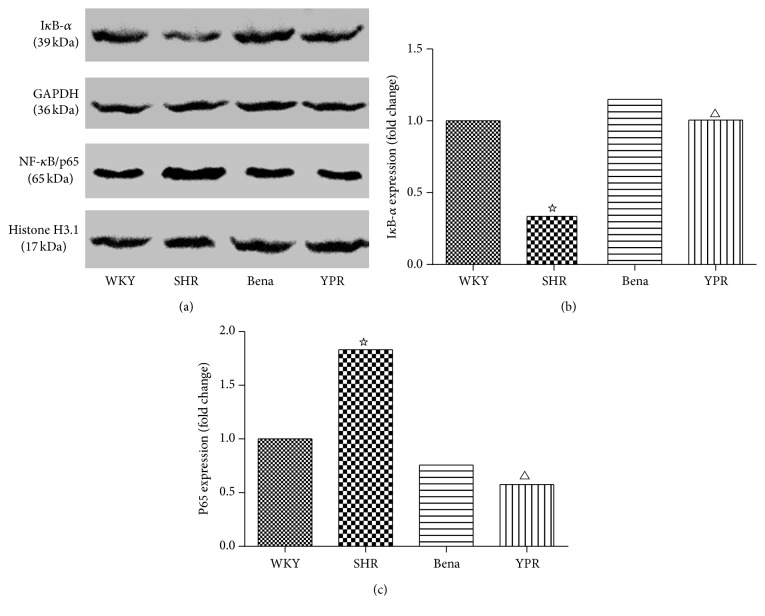
Impacts of YPR on NF-*κ*B activity in renal tissues. (a) Compared with the WKY group, in the SHR group, I*κ*B-*α* expression was significantly reduced and nuclear NF-*κ*B/P65 expression level was significantly increased, while the I*κ*B-*α* expression was not inhibited and nuclear NF-*κ*B/P65 expression was not increased after the Bena and YPR were administered, indicating that YPR could inhibit the nuclear translocation of NF-*κ*B/P65 and degradation of I*κ*B-*α*. (b) I*κ*B-*α* expression: compared with the WKY group ^☆^ *P* < 0.01; compared with the SHR group ^△^ *P* < 0.01. (c) NF-*κ*B/P65 expression levels: compared with the WKY group ^☆^ *P* < 0.01; compared with the SHR group ^△^ *P* < 0.01.

It has been demonstrated that, as compared with WKY rats, the activity of NF-*κ*B in renal tissues was significantly increased in SHR rats [11] and the expressions of cytokines including IL-1*β*, IL-6, and TNF-*α* were considerably increased [12, 13]. The inhibition of NF-*κ*B could reduce the level of inflammatory cytokines [14–16]. It has been demonstrated that increasing the cytokines in renal tissues of SHR rats was related to the activation of NF-*κ*B. The results above indicated that YPR could inhibit the expressions of inflammatory cytokines in SHR renal tissues. Thus, we detected whether these effects were related to NF-*κ*B. Results showed that, compared with WKY rats, the activity of NF-*κ*B in SHR renal tissues was significantly increased, manifested as I*κ*B degradation and NF-*κ*B p65 nuclear translocation increasing. YPR could not only inhibit I*κ*B degradation but also inhibit NF-*κ*B p65 nuclear translocation, indicating that it could inhibit the NF-*κ*B signaling (Figure 4). This effect could be one of the mechanisms of YPR reducing inflammatory cytokines expressions in SHR renal tissues.

## 4. Discussion

This study showed that YPR could significantly reduce renal damage in SHR and inhibit the expression of inflammatory cytokines including IL-1*β*, IL-6, and TNF-*α*. This effect could be associated with its inhibition of NF-*κ*B signaling activation.

It has been suggested that YPR could not only lower the blood pressure of the patients with primary hypertension but also significantly reduce microalbuminuria in patients [6]. It could improve the glomerular high-pressure, high-perfusion, and high-filtration status, regulate renal blood disorder, and improve renal arteriole spasm, sclerosis, and glomerular capillary sclerosis [6, 17]. However, the mechanisms by which YPR protects renal function remain unclear. In this study, the blood pressure of 24-week-old SHR rats was significantly higher than that of WKY rats and urine mAlb, *α*1-MG increased significantly with renal morphological changes, indicating that the damaged renal function in SHR YPR treatment could significantly inhibit the pathological changes described above, suggesting its renoprotective effects on SHR.

The kidney damage in hypertension is related to many factors. It has been found in a number of studies in recent years [18, 19] that, in renal tissues under high blood pressure, there is a great number of inflammatory cells infiltrating and the abnormal expression of inflammatory cytokines such as TNF-*α*, IL-6, and IL-1*β* increases [20, 21], indicating that inflammation reaction is one of the important factors in the occurrence of hypertensive renal damage. This study revealed that YPR could inhibit the expressions of inflammatory cytokines in SHR rats kidney, including TNF-*α*, IL-6, and IL-1*β*, suggesting that YPR could ameliorate the inflammation status.

NF-*κ*B plays a central regulatory role in tissue and organ damage mediated by numerous inflammatory factors and cytokines [22]. The major mechanism of NF-*κ*B activation is increased protein phosphorylation degradation of I*κ*B-*α*. Thus, the free NF-*κ*B translocated into the nuclear is increased [23]. NF-*κ*B is widely presented in glomerular endothelial cells, epithelial cells, and renal tubular cells. NF-*κ*B signaling activation could significantly damage the kidney [24], and the mechanism is related to the expression of inflammatory cytokines mediated by NF-*κ*B. In addition, the inhibition of NF-*κ*B signaling pathway could reduce inflammation [15] and improve renal functions [25]. Since YPR could inhibit the levels of inflammatory cytokines, such as TNF-*α*, IL-6, and IL-1*β*, we further detected whether these effects were related to NF-*κ*B. It has been displayed in the study that, compared with WKY rats, NF-*κ*B p65 nuclear translocation and I*κ*B degradation were significantly increased in SHR rats, indicating the activated NF-*κ*B signaling in SHR. YPR could inhibit the NF-*κ*B signaling activation, which might be one of the mechanisms in YPR inhibiting inflammatory cytokines and protecting the kidney.

## 5. Conclusion

In summary, YPR could improve renal function of SHR and inhibit the expression of inflammatory cytokines, which may be attributed to its inhibition of NF-*κ*B signaling.

## Figures and Tables

**Table 1:** Blood pressure in each group.

	*N*	Systolic blood pressure (SBP)	Diastolic blood pressure (DBP)
WKY group	7	149.08 ± 3.68	111.95 ± 5.62
SHR group	7	196.96 ± 3.77^##^	146.08 ± 4.82^##^
Bena group	7	158.53 ± 3.79^#△^	117.61 ± 1.71^#△^
YPR group	7	161.45 ± 7.57^#△^	117.21 ± 5.17^#△^

Compared with WKY group, ^##^ *P* < 0.01, ^#^ *P* < 0.05; compared with SHR group, ^△^ *P* < 0.01.
